# Commercial immunoassays in paraneoplastic neurological syndromes: an Australian laboratory perspective

**DOI:** 10.3389/fneur.2025.1515069

**Published:** 2025-02-14

**Authors:** Syed B. Ali, Amelia Cecchin, Rebecca Burfoot, Nicholas Chia, Janakan Ravindran, Deborah Field, Jovanka King, Phillippa A. Pucar, Tatjana Banovic

**Affiliations:** ^1^Department of Clinical Immunology and Allergy, Flinders Medical Centre, Bedford Park, SA, Australia; ^2^School of Medicine and Biomedical Sciences, Flinders University, Bedford Park, SA, Australia; ^3^Department of Clinical Immunology and Allergy, Royal Adelaide Hospital, Adelaide, SA, Australia; ^4^School of Medicine and Biomedical Sciences, University of Adelaide, Adelaide, SA, Australia; ^5^Department of Immunopathology, SA Pathology, Adelaide, SA, Australia; ^6^Department of Neurology, Royal Adelaide Hospital, Adelaide, SA, Australia; ^7^Department of Clinical Immunology and Allergy, Women’s and Children’s Hospital, Adelaide, SA, Australia

**Keywords:** neuronal immunoblot, paraneoplastic neurologic syndromes, neuronal antibodies, indirect immunofluorescence, immunoassay

## Abstract

**Background:**

Paraneoplastic antibodies are implicated in heterogeneous clinical presentations. Commercial immunoassays include indirect immunofluorescence (IIF), and line immunoblot (LIB). LIB can be associated with false positives, and unfortunately, further confirmatory assays are not readily available in diagnostic laboratories.

**Objectives:**

To determine frequency of positive LIB on serum or cerebrospinal fluid (CSF) using EUROLine paraneoplastic neurological syndromes (PNS) 12 Ag Test kit (EuroImmun, Germany) and establish concordance with IIF on Nova Lite kit (Inova Diagnostics, United States) and clinical presentation.

**Methods:**

A retrospective analysis of all LIB performed over a four-year period was undertaken. Healthy control samples were also analysed with IIF and LIB.

**Results:**

Two thousand and eighty-one LIB samples were processed, 91 (4.4%) were positive from 69 patients with a median age of 64 years. There were 37 females (53.6%). Some samples had two antibody specificities (*n* = 6, 6.6%). Of those with one antibody, GAD65 (*n* = 22), Yo (*n* = 19), SOX1 (*n* = 17) and amphiphysin (*n* = 14) were most frequent. Of the positive LIBs, 80 (87.9%) had concurrent IIF and eight samples (10%) had a typical IIF pattern. Clinical relevance of a positive LIB, irrespective of IIF, was seen in 15/91 samples (14.3%) from nine patients; GAD65 (*n* = 3), Hu (*n* = 2), amphiphysin (*n* = 1), Yo (*n* = 1), Tr (*n* = 1) and CV2 (*n* = 1). Of the 71 healthy controls, five (7.0%) had a positive LIB: medium band (*n* = 4, 5.6%: amphiphysin, CV2, SOX1 and Yo) and strong band (*n* = 1, 1.4%: Yo). All IIF were negative. On average, signal intensity (SI) was higher in those with disease (SI 77.3/very strong band) compared to those without (SI 28.6/strong band) and healthy controls (SI 2/negative band) (*p* < 0.0001).

**Discussion:**

LIB has a high false positive rate, and in this cohort, there were more false than true positive results. The assay must be used in those with a high clinical suspicion for PNS. While the commercial IIF kit is a useful test, it is insufficient to be used as a screening strategy in isolation.

## Introduction

Paraneoplastic neurological syndromes (PNS) are immune-mediated diseases of the nervous system that occur as an indirect effect of malignancy, often in the presence of antibodies (1). The role of such antibody testing is increasingly being recognised in clinical care for patients presenting with heterogeneous neurological presentations such as encephalitis, polyneuropathy, and seizures. Recently, the variable association of these antibodies with malignancy has been acknowledged, and this is reflected in the diagnostic criteria which recommends the terms “low-, intermediate- and high-risk neuronal antibodies,” depending on the strength of the malignancy association (2).

The recommended method for detection of PNS antibodies which are targeted against intracellular antigens, is by a screening immunohistochemistry (IHC) or indirect immunofluorescence (IIF) performed on rodent brain tissue substrate, followed by a confirmatory assay such as line immunoblot (LIB) (3). Both IHC and IIF require freshly prepared rodent brain tissues with specific processing protocols, which are costly and labour intensive; hence impractical in a diagnostic laboratory (4). Commercial IIF kits are available to circumvent these problems and use primate and rodent tissue substrates. However, not all these antibodies have a specific IIF pattern on the kits, namely recoverin and titin antibodies. Other confirmatory assays include western blot (WB) and transfected cell-based assay (CBA), which are primarily used in highly specialised neuroimmunology laboratories (5).

PNS LIBs are available through various manufacturers and are easy to apply in diagnostic laboratories. Although they are more sensitive in the detection of low levels of paraneoplastic antibodies than IHC, they are also less specific for PNS (3). Therefore, false positive results are problematic, particularly if driven by indiscriminate testing. This can lead to serial malignancy screening, resulting in an emotional burden to the patient and a significant financial implication to healthcare (6, 7). Furthermore, a very weak or low positive LIB result often has little clinical significance, especially when other supportive methods such as IHC or IIF are negative (4). Given PNS is rare, with a prevalence of 4/100,000 person-years, no recommended cut-offs currently exist for a positive LIB as this can differ depending on the PNS antibody (8).

## Objectives

The three objectives of this study were firstly to retrospectively assess the frequency of positive paraneoplastic antibody results using LIB in patients and controls, secondly to determine concordance of positive results with clinical neurological disease, and thirdly to assess the utility of a commercial IIF product for the diagnosis of PNS.

## Methods

### Ethics

This study has been approved by the Ethics Committee at Central Adelaide Health Network (Reference Number: 18203).

### Patient samples

All positive PNS LIB processed between November 2018 and December 2021 from a tertiary immunopathology laboratory in Adelaide, South Australia were analysed. Data including patient demographics, clinical presentation, laboratory, radiology and other investigation results, diagnosis and management were collected from clinician request forms or via the electronic medical records (EMR). Patient location (either hospital in-patient or community out-patient) was collated, as well as requesting clinician specialty. If either the request form or EMR had insufficient information, data was left blank.

Serum and CSF samples were processed on PNS LIB (EUROLine PNS 12 Ag Test, EuroImmun, Germany) and IIF (Nova Lite Monkey Cerebellum/Cerebrum and Mouse stomach, Inova Diagnostics, United States) in accordance with manufacturer’s instructions. Specifically, for the LIB assays, serum and CSF were diluted to 1:10 and 1:4, respectively. For the IIF assays, sera and CSF were tested at dilutions of 1:50 and 1:1 (neat), respectively. This PNS LIB tested the following antigen specificities: amphiphysin, CV2, GAD65, Hu, Ma2/Ta, recoverin, Ri, Tr, SOX-1, titin, Yo and Zic4. Of these typical antibodies, IIF patterns are recognised for all except for recoverin and titin as these require specialised retinal tissue and striated muscle respectively, and hence were excluded (9, 10).

To exclude possible prozone effect, dilution of both serum (1:50 and 1:100) and CSF (neat and 1:10) were performed for samples with strong or very strong positive bands detected on the PNS LIB that returned negative IIF results at standard dilution.

The signal intensity (SI) of PNS LIB was determined by EUROLineScan Flatbed scanner. While patient data was reviewed for results with a very weak band, i.e., (+)/SI 6–10, these were not included in the formal analysis. All medium band +/SI 11–25; strong band ++/SI 26–50 and very strong band +++/SI >50 samples were included. Correlation of these results with IIF were documented as typical paraneoplastic antibody pattern, non-specific/atypical pattern, anti-nuclear antibody (ANA) pattern or negative. ANA staining was assessed in the cerebellum, cerebrum and stomach tissues to ensure all possible antibody specificities were excluded, for example the myenteric plexus was reviewed in the stomach to rule out anti-Hu antibodies.

Medical records of patients with positive PNS LIB results were retrospectively reviewed to assess the likelihood of an autoimmune or PNS clinical syndrome. The PNS-Care Score was calculated (2), and the inclusion of cases as “true” positive results was by consensus of three clinical neurologists (authors NC, JR, and DF) from a tertiary hospital Neurology Department in Adelaide, Australia.

### Healthy controls

To provide insights of background prevalence, a LIB and IIF was run on a group of health controls. Healthy control sera without a history of autoimmune conditions, paraneoplastic diseases or malignancy stored from a previous unrelated study were used. After initial use, these sera were immediately frozen and stored at −80°C, before being adequately thawed, spun and tested in the same manner as the patient samples with both IIF and LIB for this current project.

### Statistical analysis

Descriptive statistical methods were performed, including unpaired *t*-test statistical analysis through GraphPad Prism software (Version 9.5.1). Univariate comparison of categorical variables was performed using Fisher’s exact test and a *p*-value of <0.05 was considered significant.

### Data availability

All data related to this study is anonymized, and data not published within this article will be made available by request from a qualified investigator.

## Results

### Summary of LIB results

**Figure 1:**
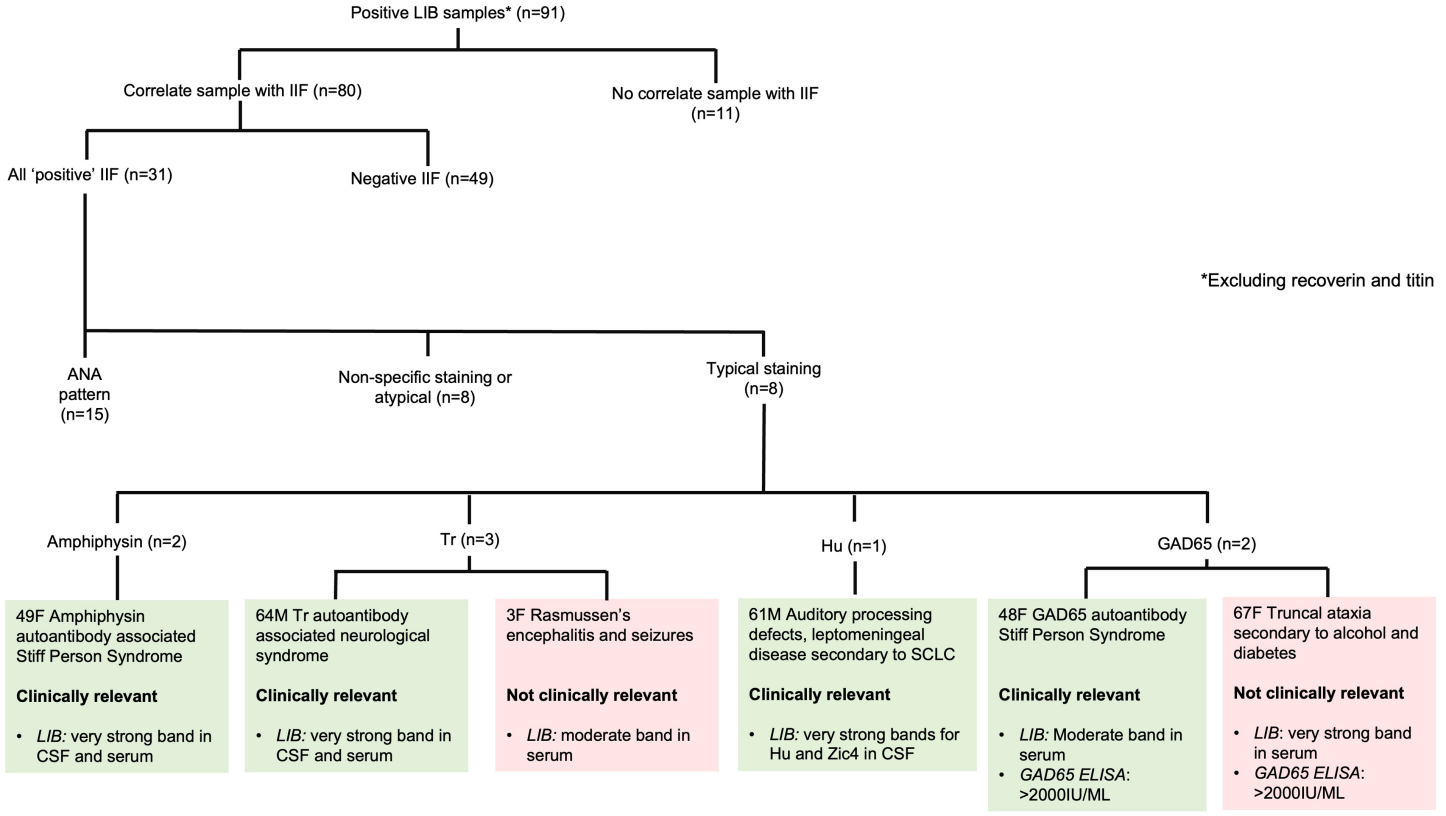
Correlation of positive paraneoplastic neurological syndromes line immunoblot results with IIF. ANA - antinuclear antibody, ELISA - enzyme-linked immunosorbent assay, LIB - line immunoblot, SCLC - small cell lung carcinoma, SPS - Stiff Person Syndrome.

Of the 2,081 PNS LIB resulted, 139 (6.7%) were positive for neuronal antibodies by LIB. When recoverin and/or titin antibodies were excluded (*n* = 48, 2.3%), 91 (4.4%) were included for analysis (Figure 1). The remainder were negative (*n* = 1,942, 93.3%); either a very weak band (*n* = 61) or no signal on LIB (*n* = 1,881). The collective 91 samples were from 69 patients; both serum and CSF (*n* = 16); serial serum (*n* = 3), serum only (*n* = 35), and CSF only (*n* = 15).

Most of the positive LIB from 69 patients were from hospitalised patients (*n* = 54, 78.3%), while the remainder were community outpatients (*n* = 15, 21.7%). Neurologists were the primary requesting clinicians (*n* = 45, 65.2%) followed by general medicine (*n* = 16, 23.2%), other medical specialties (*n* = 5, 7.2%), and psychiatry (*n* = 3, 4.3%). The number of positive LIB requests increased over the four-year period: 2018, *n* = 5, 2019, *n* = 4, 2020, *n* = 21, and 2021, *n* = 39 (Table 1).

**Table 1:** Case series demographics of the patients with positive line immunoblots.

Total patients: *n* = 69	
Age	Range: 3–88 years Mean: 58.8 years Median: 64 years
Gender	37 females (53.6%) 32 males (46.4%)
Requesting year	2018: 5 2019: 4 2020: 21 2021: 39
Requesting location	Hospital inpatient: 54 (78.3%) Outpatient/specialist clinic: 15 (21.7%)
Requesting speciality	Neurology: 45 (65.2%) Non-neurology: 24 (34.8%) General medicine: 16 (23.2%) Medical specialties: 5 (7.2%) Haematology: 1 Oncology: 1 Infectious disease: 1 Respiratory: 1 Ophthalmology: 1 Psychiatry: 3 (4.3%)

### Positive LIB antibody specificities

The most common positive PNS LIB antibody specificities were GAD65, Yo, SOX1 and amphiphysin. There were no Ri antibody positive samples in the cohort. The positive antibody results, including SI values are listed in Table 2. Six patients had more than one antibody detected in their serum and/or CSF. All patients who had LIB results for both CSF and serum (*n* = 34) had concordant antibodies identified.

**Table 2:** Descriptive results of the autoantibody specificities and neuronal immunoblot signal intensity of the patients with positive line immunoblots.

	Amphiphysin (*n* = 14)	CV2 (*n* = 5)	GAD65 (*n* = 22)	Hu (*n* = 5)	Ma2/Ta (*n* = 3)	SOX1 (*n* = 17)	Tr (*n* = 6)	Yo (*n* = 19)	Zic4 (*n* = 4)
Minimum	11	11	11	32	13	11	11	11	11
Maximum	159	21	122	143	30	53	155	69	90
Median	21.5	17	20	66	24	17	22	22	43.5
IQR (25, 75)	14, 120.5	12.5, 20.5	12.8, 54.5	36.5, 136	13, 30	15, 23	11, 136.3	13, 34	16.3, 81.3

IQR, interquartile range (1st quartile, 3rd quartile).

### Confirmation of LIB with IIF pattern

Of the 91 positive LIB samples, IIF was performed in 80 (87.9%) samples. A typical pattern of staining (consistent with the antibody identified on LIB) was present in eight (25.8%) samples: amphiphysin (two samples from one patient), Tr (three samples from two patients), Hu (one sample), and GAD65 (two samples from two patients). The remaining 72 samples had either ANA or non-specific/atypical staining or were negative (Figure 1).

There were eight stored samples with strong/very strong positive LIB and negative IIF on which serial dilutions in serum and CSF were negative and excluded the prozone effect (Supplementary Figure S1).

### Clinical details of positive LIB results

Autoimmune or paraneoplastic neurological syndromes were confirmed in 15/91 (16.4%) LIB positive samples from nine patients. Six patients had both CSF and serum samples (66.7%), two serum alone (22.2%), and one CSF alone (11.1%). Except for one patient with LIB positivity for two antibodies (Hu and Zic4), the remaining patients all had a single antibody identified on LIB.

Table 3 outlines a case summary of patients with “true positive” LIB results, as well as patients who had discordant clinical findings compared to LIB and IIF and hence were considered to be “false positive” results. Five patients were deemed to have “definite” paraneoplastic neurological disorders (PNS-Care Score 9–10). One patient presenting with myeloneuropathy with CV2 antibodies on LIB without typical staining on IIF and for whom no malignancy was identified at follow-up was included as a “probable” paraneoplastic neurological disorder (PNS-Care Score 6). Another patient with CSF GAD65 antibodies by LIB but not IIF presented with subacute cognitive change and aphasia simultaneous to uterine serous carcinoma. She had CSF pleocytosis with rapid response to immunotherapy—while the pathogenicity of the GAD65 antibodies is uncertain and only criteria for “possible” paraneoplastic disorder were met, she was included as a true positive by consensus. Lastly, there were two patients with GAD65 antibody positive stiff person spectrum disorder who were deemed true cases of autoimmune (non-paraneoplastic) neurological disease.

**Table 3:** Definite cases with clinical features correlating to autoantibodies including presentation, methods of detection: CSF (C) or serum (S), diagnosis, treatment, and history of malignancy.

Case presentation	Modality of testing	Diagnosis	Ancillary tests	Treatment	Malignancy	PNS-Care Score
39 M myeloneuropathy	S: IIF: negative; LIB: CV2—moderate positive (20) C: IIF negative; LIB: CV-2—moderate positive (11)	Autoimmune myelitis secondary to CV-2 autoantibody	CSF: IgG: albumin 16%, OCB not detected MRI: T2 hyperintensity—dorsal columns spinal cord with mild spinal cord enlargement PET: negative	PLEX, corticosteroids, and rituximab	Negative	6
64 F subacute non-length-dependent sensory change	S: IIF: ND, LIB: Hu—very strongly positive (129) C: ND	Anti-Hu antibody associated subacute sensory neuronopathy	CSF: IgG: albumin 23%, OCB N/A EMG: non-length-dependent sensory neuropathy	IVIG	Metastatic lung adenocarcinoma	10
64 M spinocerebellar syndrome and complex ophthalmoplegia	S: IIF: Tr staining, LIB: Tr—very strong positive (130) C: IIF: Tr staining, LIB: Tr—very strong positive (155)	Anti-Tr antibody associated neurological syndrome	CSF: IgG: albumin 12% MRI brain and spine: normal CT: bilateral iliac and inguinal adenopathy	Corticosteroids and chemotherapy	T-lymphoproliferative disorder	9
77 F cerebellar ataxia	S: IIF: ND, LIB: Yo very strong positive (69) C: ND	Anti-Yo antibody associated PNS	MRI: progressive cerebellar atrophy No CSF performed	PLEX and corticosteroids as well as debulking surgery with chemotherapy	Ovarian carcinoma	10
49 F spasticity, lower limb weakness and muscular spasms	C: IIF: amphiphysin staining LIB: amphiphysin very strong positive (159) S: IIF: amphiphysin staining LIB: amphiphysin very strong positive (143)	Anti-amphiphysin antibody associated SPSD	CSF: IgG: albumin 33%, protein 0.58 g/L, OCB—detected MRI brain and spine: normal PET scan: negative	Corticosteroids	Breast carcinoma	9
71 F progressive mixed receptive and expressive dysphasia	C: IIF: ANA pattern; LIB: GAD65 moderate positive (21) S: IIF: ANA pattern LIB: GAD65—moderate positive (11)	Anti-GAD65 antibody autoimmune encephalitis	CSF: IgG: albumin ratio 33%, protein 0.41 g/L, OCB—detected MRI brain: negative PET scan: metastases—adrenal and distal nodal	Corticosteroids, IVIG, surgical debulking and chemotherapy	Endometrial carcinoma	4
61 M subacute hearing loss, pontine gaze paresis and ataxia	C: IIF: Hu-like pattern; LIB Hu very strong positive (143), Zic4 very strong positive (55) S: ND	Anti-Hu associated PNS	CSF: IgG: albumin ratio 16%, protein 1.47 g/L, OCB—detected MRI brain: nil significant CT scan: right hilar mass and pulmonary nodule	Short corticosteroid trial^a^	Small cell lung carcinoma	9
49 F lower limb stiffness and abnormal gait	C: IIF: ND, LIB: GAD65 very strong positive (58) S: IIF: Negative and LIB: moderate positive	Anti-GAD65 antibody SPSD	CSF: IgG: albumin 16%, protein 0.34 CT: negative MRI: negative	Corticosteroids and IVIG	Negative	2
48 F whole-body stiffness, hyperekplexia	C: IIF ND, LIB GAD65—moderate positive (26) S: IIF negative LIB: GAD65—strong positive (54) ELISA: >2,000 U/mL	Anti-GAD65 antibody SPSD	CSF: IgG: albumin: ND, protein 0.25 g/L	Corticosteroids and IVIG	Negative	2

ND, not done; IIF, indirect immunofluorescence; IVIG, intravenous immunoglobulin; SPSD, stiff person spectrum disorder; PLEX, plasma exchange; OCB, oligoclonal bands, CSF protein range 0.15–0.45 g/L, IgG: albumin ratio range 0–12%.

a Deceased.

Two patients with antibody positivity on LIB with confirmation on IIF did not have a compatible neurological clinical syndrome. The first was a 3-year-old female with a diagnosis of Rasmussen’s encephalitis positive for Tr antibodies. The second was a 67-year-old female with chronic length-dependent sensorimotor neuropathy related to diabetes and alcohol use who tested positive for GAD65 antibodies, likely related to her diabetes rather than GAD65 antibody-associated neurological autoimmunity. Neither had malignancy.

### Testing predictors of confirmed neurological syndrome

**Figure 2:**
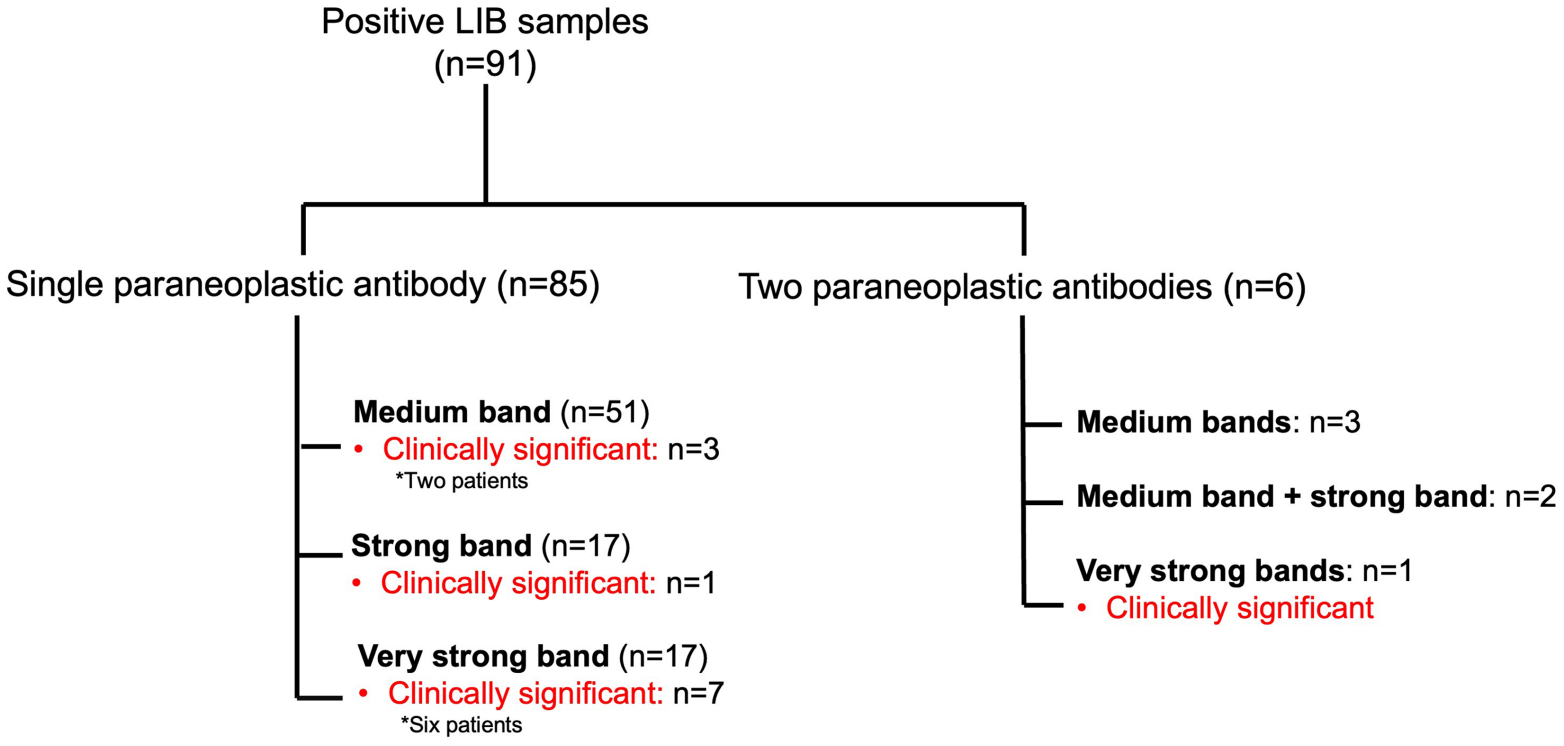
Summary of the positive paraneoplastic neurological syndromes line immunoblot samples, with band intensity and presence of clinical concordance.

The majority of the positive LIB samples had a single paraneoplastic antibody (*n* = 85, 93.4%) with the remaining having two antibodies (*n* = 6, 6.6%) (Figure 2). In the latter, these included medium bands (*n* = 3): SOX1 (SI 11) and GAD65 (SI 11), SOX (SI 11) and GAD65 (SI 13) and Tr (SI 11) and GAD65 (SI 17); medium band + strong band (*n* = 2): GAD65 (SI 12) and Hu (SI 32), and amphiphysin (SI 22) and Yo (SI 35); an very strong bands (*n* = 1): Zic4 (SI 55) and Hu (SI 143). LIB positivity of a single antibody (11/85, 12.9%) compared to two antibodies (1/6, 16.7%) did not predict the presence of a confirmed neurological clinical syndrome, *p* = 0.58. The average SI was significantly higher in those with clinical relevance (SI 77.3/very strong band) compared to those without (SI 28.6/strong band range) (*p* < 0.001) (Supplementary Figure S2). LIB tests with very strong signal intensity were more likely to be associated with a confirmed neurological syndrome in comparison to those with medium or strong SI (44.4% versus 5.5%, *p* < 0.001). Confirmed clinical diagnosis was also more likely when the LIB antibody positivity was confirmed on IIF compared to those with negative IIF testing (75% versus 5.8%, *p* < 0.01).

### Healthy controls

Healthy control serum (*n* = 71) was run on the LIB; average SI was 2.2. There were five positive (7.0%) LIB; four within the medium band range: amphiphysin (SI 15), CV2 (SI 14), SOX1 (SI 16) and Yo (SI 14) and one in the strong band range: Yo (SI 49). All five samples had negative IIF. Only age ranges and gender data were available for these samples; the median age range within this group was 36–50 and the majority were female (*n* = 61, 85.9%).

## Discussion

### False positive LIB results were common

This study demonstrates that very few positive LIBs have a clinical neurological disease correlate. Positive LIB results were seen in only 4.4% which is similar to other studies of paraneoplastic neuronal immunoblots (2). Notably, clinically relevant neurological syndromes were seen only in 15 samples obtained from nine patients, all referred by neurologists, accounting for 0.7% of all results or 16.4% of positive LIB results, respectively. This cohort’s positive predictive value (PPV) of 14.2% is lower compared to 39 and 43% reported by others (6, 11), which may be due to indiscriminate testing, particularly given the high proportion (34.8%) of requests from clinicians in fields other than neurology and relevance of the clinical details.

Over the four-year period, there was a growing number of positive results, driven in part by increased awareness of the test. Around 20% of patients referred for testing were under general medicine. The clinical significance of very weak bands detected in patient samples (*n* = 61) was also evaluated, none of which had any correlation. Hence, strategies to reduce inappropriate testing is further education. In addition, better physician education about limitations of LIB in testing would be useful, as without a clinical index of suspicion, a positive result may not be clinically relevant in a large proportion of cases. Furthermore, non-discriminatory testing will also lead to economic constraints. The paraneoplastic panel contains a number of antibodies that cause unrelated syndromes, and some laboratories are moving away from a “paraneoplastic panel” to a phenotype-specific panel (12).

The identification of false positive PNS LIB results is critical and has recently been highlighted in the updated diagnostic criteria for PNS (2, 13, 14). Firstly, some of the antibodies in this panel have a known higher false positive rate than others, such as Yo, Ma2, CV2 and SOX1 antibodies (2, 15). This is reflected in this cohort, in which false positive Yo and SOX1 antibodies were prevalent, accounting for 34% of all false positive LIB results (all were negative on IIF). Similarly, two healthy controls had positive Yo antibodies detected in LIB without concordant IIF. False positive Yo antibody results on LIB have been increasingly recognised, with published case series reporting a rate between 70 and 85% (4, 16). While LIB contains the paraneoplastic cerebellar degeneration related antigen (CDR2), the main antigen implicated in Yo antibody related disease is CDR2L, which only shares 45% sequence homology with CDR2 (17–19). This may account for the high false positive rate observed in the study, and suggests that only positive results with very strong bands are likely to be clinically relevant (3). Secondly, PNS antibodies have been reported to have a high background prevalence of up to 2% in the general population, which supports that requesting those with higher pre-testing probability of disease is of utmost importance (11, 20, 21). In our cohort, the PPV of 14.2% would indicate that a positive LIB result indicates 7.1 times higher likelihood of not having a PNS, hence the need for more discriminative testing.

### Test characteristics may assist interpretation of positive results

**Figure 3:**
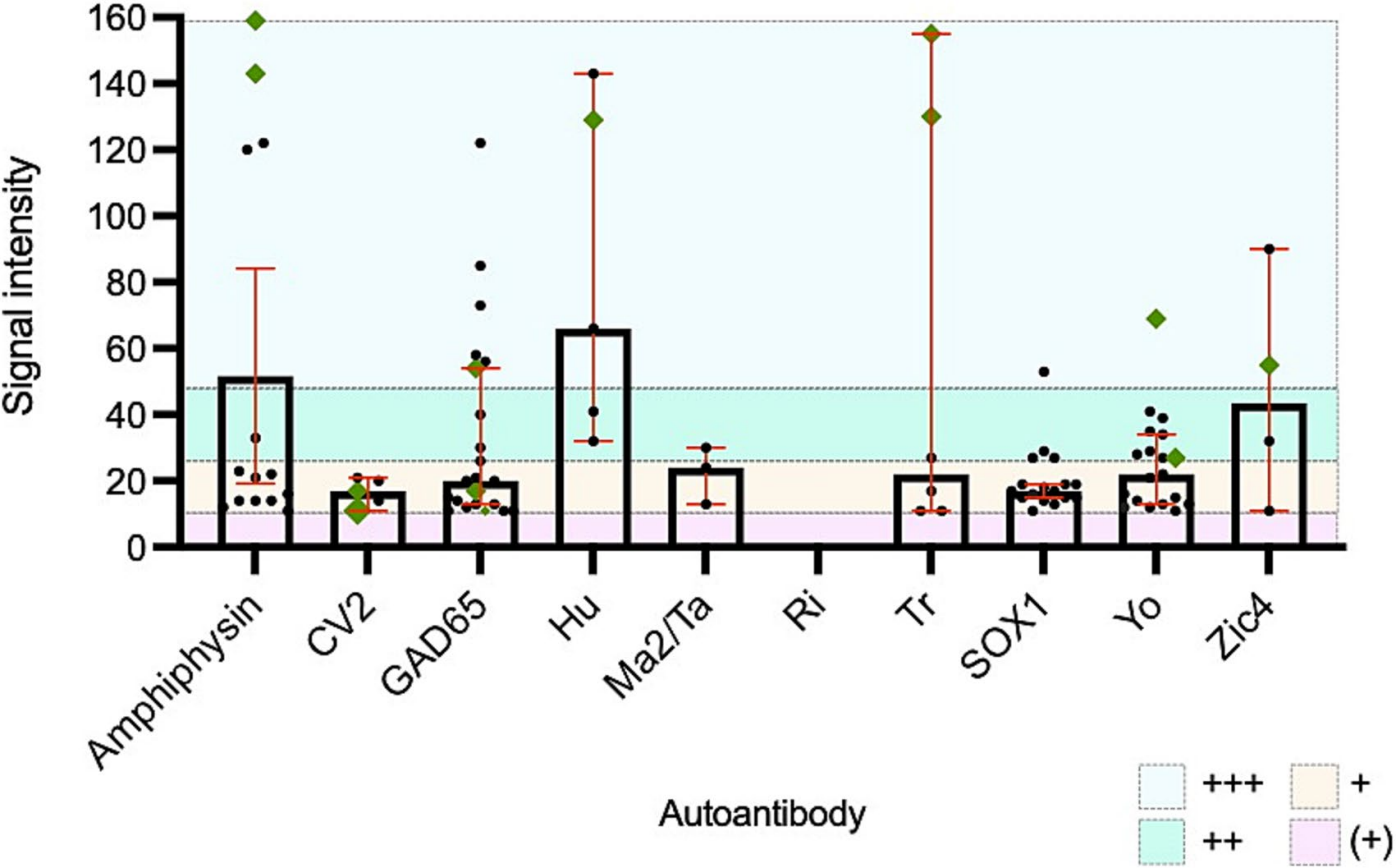
All positive paraneoplastic neurological syndromes line immunoblot results with confirmed clinical concordance. Green diamonds represent cases with confirmed relevant neurological disease. +++: very strong positive band/signal intensity >50, ++: strong positive band/signal intensity 26-50, +: moderate positive band/signal intensity 11–25 and (+): weak positive band/signal intensity 6–10.

Establishing optimal SI cut-offs is challenging but has the potential to increase the certainty of the clinical relevance of a positive result. In this cohort, positive LIB results with an SI of “very strong” were more likely to indicate true underlying neurological autoimmunity (in comparison to moderate or strong SI). This was particularly notable for amphiphysin, Hu and Tr antibodies, but was not apparent with Yo antibodies (Figure 3). Prior studies have demonstrated that optimal SI cut-offs likely vary between different PNS antibodies. Hu and Yo antibodies were only confirmed to be relevant in those with “very high” SI, whereas other antibodies were occasionally relevant at lower SI cut-offs (4). This highlights the complexities with PNS LIB SI interpretation and further studies of commercial LIB products are required to define optimal cut-off points for each reported antibody. On the other hand, weak positive LIB results are likely irrelevant and should be interpreted with caution (3, 4). In this cohort, none of the 61 samples with single antibodies with SI in the “very weak” band had a confirmed PNS.

Paired CSF and serum samples are recommended to maximise clinical specificity, rate of antibody detection and the identification of possible false positive results (2, 5, 15, 22). In this cohort, all 34 paired CSF had concordant serum antibodies detected.

### Commercial IIF kits have utility, but may have lower sensitivity

Recently, emphasis on the requirement of further confirmation by tissue IHC or IIF following a positive PNS LIB result for intracellular PNS autoantibodies has been reported due to its ability to detect a broad range of antibody staining patterns (“hypothesis free”) and higher sensitivity and specificity for some antibodies likely due to detection of specific epitopes (2, 3, 23). Commercial IIF kits are easily available, however their diagnostic performance in comparison to the custom in-house IIF testing that has established the current literature is not known. In this study, the Nova Lite IIF was useful as a confirmatory diagnostic test in most patients with compatible autoimmune or paraneoplastic neurological disorders, and concordant positivity on LIB and commercial IIF increased the likelihood of a true positive result compared to LIB positivity with discordant IIF (odds ratio 31.1). A prior study found concordance of commercial LIB with frozen rat cerebellar IHC in 48% of cases as well as a much higher proportion of clinically relevant antibody results (3). This could relate to higher sensitivity of their in-house IHC compared to the Nova Lite IIF, although selection bias likely had an influence as a portion of their cases were included based on a positive IHC study.

IHC and fresh tissue IIF samples are not routinely performed in all diagnostic laboratories due to several factors including increased time and labour required for preparing the rodent tissue and associated animal ethics application required to obtain the tissue, and the specific expertise required in interpreting positivity and antibody specificity relating to the tissue substrate (24, 25). Furthermore, as the test interpretation is more subjective, some cases may be missed. In our laboratory, IIF slides are read by two trained scientists, and if the readings are discordant, a third reader is involved to address any degree of operator dependent subjectivity (5). An immunopathologist is consulted prior to reporting any non-specific patterns. Additionally, quality assurance program (QAP) samples are processed quarterly to ensure optimal performance. Finally, while the prozone effect has been recognized in IIF (26), this was not the case in the eight samples that were tested.

### Laboratory workflow in processing samples

While diagnostic confirmation with a second test is generally recommended, there is limited data on whether simultaneous or sequential testing is more sensitive or specific, and whether one is more cost effective. In our laboratory, the testing algorithm involves performing IIF first, and positive samples are then tested using LIB. However, if the clinician specifically requests LIB, then both immunoassays are performed concurrently. One study assessing the diagnosis of Ma2 antibody PNS demonstrated that simultaneous testing did not improve diagnostic accuracy in comparison to sequential testing (23). However, IIF was performed using fresh tissue and further testing with WB and CBA were performed in cases with discrepant results (23). This study selected patients for IIF testing based on a positive LIB result, therefore it is unknown if some patients with unidentified PNS may have had IIF positivity in the absence of positive LIB. Collectively, the performance of the commercial IIF kits as an initial diagnostic test when screening for PNS requires further evaluation. A prospective study with large numbers of patients evaluating both these assays would be helpful to identify whether the NOVA Lite IIF has limited diagnostic utility. Ideally, this would include collection of a well-phenotyped cohort in which all patients are assessed using LIB and IIF or specialised neuroimmunology laboratories evaluating commercially available IIF kits compared to their own in-house methods.

### Study limitations

Our study had several limitations which need to be considered. Firstly, further assays such as fresh tissue IIF, WB or CBA were not performed in those with a negative commercial kit IIF and positive LIB, which may have further supported true positive results. Secondly, the clinical confirmation of a PNS was made retrospectively. Some of the privately referred outpatients had incomplete clinical details on request forms, while for the remainder of patients, clinical details were obtained through review of medical records. Consensus was obtained amongst three neurologists, who in most cases were not involved in the patients’ care. Thirdly, GAD65, which is tested as part of the 12 antigen PNS panel is not typically associated with malignancy but has been included in this study primarily to determine concordance of antibody detection with clinical phenotype. Overall, the rate of concordance between IIF and LIB may be underestimated in those with a PNS diagnosis, as not all samples were tested by IIF. Fourthly, other PNS LIB were not used for comparison, and hence generalisability to all LIB kits may not be applied. Fifthly, we did not have detailed clinical information about the cohort of LIB negative, meaning that potentially some patients with true PNS may have been missed. Lastly, a large proportion of the healthy control sera was collected from females, which may not address some antibody specificities, e.g., Ma2 antibodies which are associated with testicular cancer.

## Concluding statements

A positive LIB result can be useful in the diagnostic work up of suspected PNS, when accompanied by ancillary tests and a high clinical suspicion. However, as this study demonstrates, indiscriminate testing leads to a very large proportion of positive results that may lead to diagnostic confusion, patient distress, and unnecessary and costly serial serological, CSF and malignancy screening. Very weak positive bands in PNS LIB are likely false positives, whereas at least for some antibodies a “very strong” positive is more likely to be clinically relevant. In centres that do not have referral access to highly specialised neuroimmunology laboratories, commercial IIF kits are feasible to deploy in the immunology laboratory and are a promising confirmatory test after the widely-available LIB to improve diagnostic specificity. More study is needed to determine if commercial IIF kits have sufficient sensitivity for use as a primary screening method.

## Data Availability

The raw data supporting the conclusions of this article will be made available by the authors, without undue reservation.

## Supplementary material

SUPPLEMENTARY FIGURE S1 Prozone effect assessment example in a patient with positive Zic4 antibody on paraneoplastic neurological syndromes line immunoblot (Signal Intensity 32; ++ strong positive band) and CSF (Signal Intensity 90; +++ very strong positive band). CSF dilution (1:10) with indirect immunofluorescence findings for cerebellum **(a)**, cerebrum **(b)** and stomach **(c)** tissue substrates. Serum dilution (1:100) with IIF findings for cerebellum **(d)**, cerebrum **(e)** and stomach **(f)** tissue substrates. CSF - cerebrospinal fluid.

SUPPLEMENTARY FIGURE S2 Signal intensity of positive paraneoplastic neurological syndromes line immunoblot results in patients with or without clinical correlation as well as healthy controls. *****p* < 0.0001.
