# Supplementary figures and images for: Commercial immunoassays in paraneoplastic neurological syndromes: an Australian laboratory perspective
Source: Front Neurol. 2025 Feb 14;16:1515069. doi: 10.3389/fneur.2025.1515069 (PMC11868762; doi:10.3389/fneur.2025.1515069)

## Slide 1
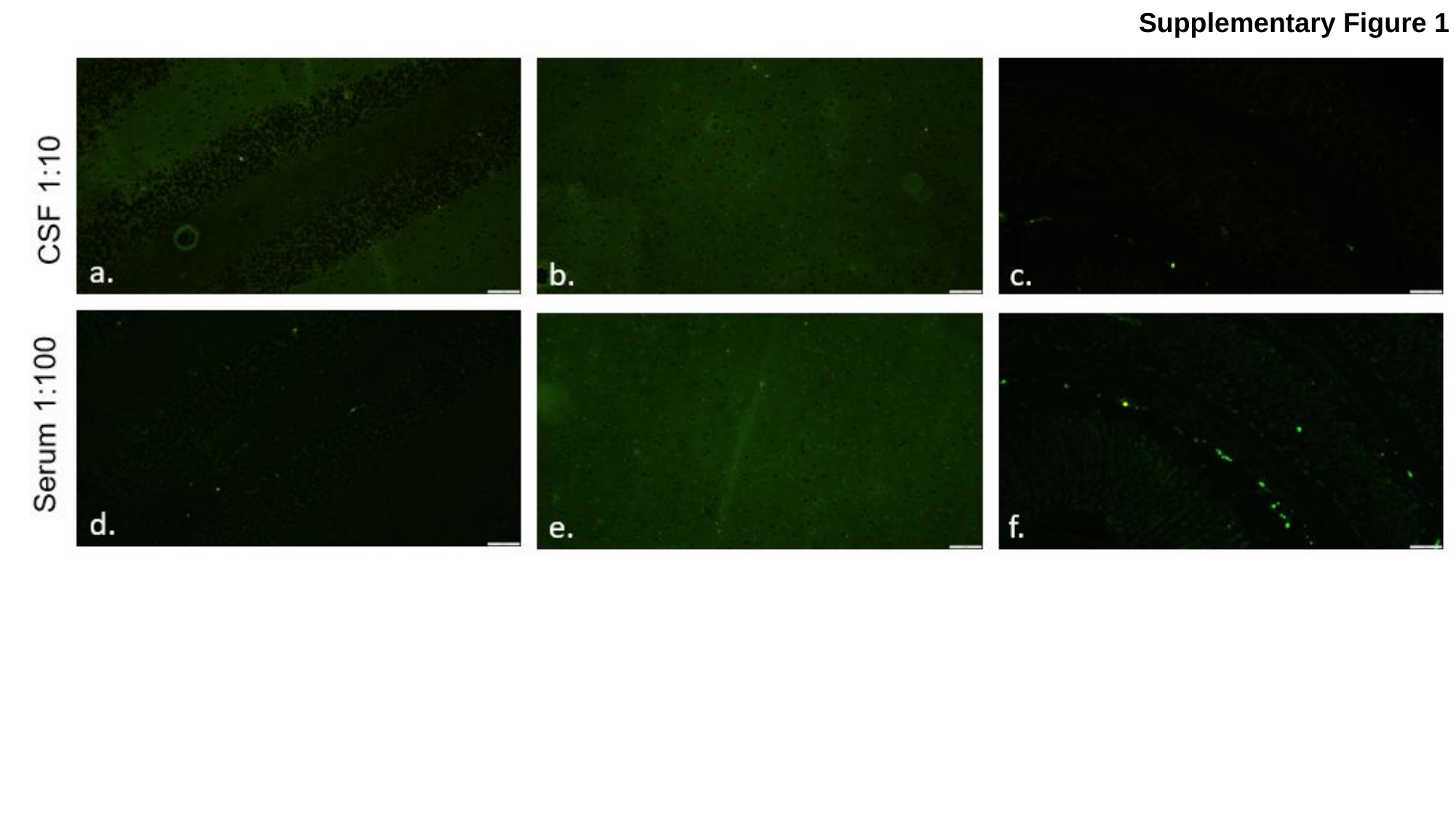

Supplementary Figure 1

## Slide 2
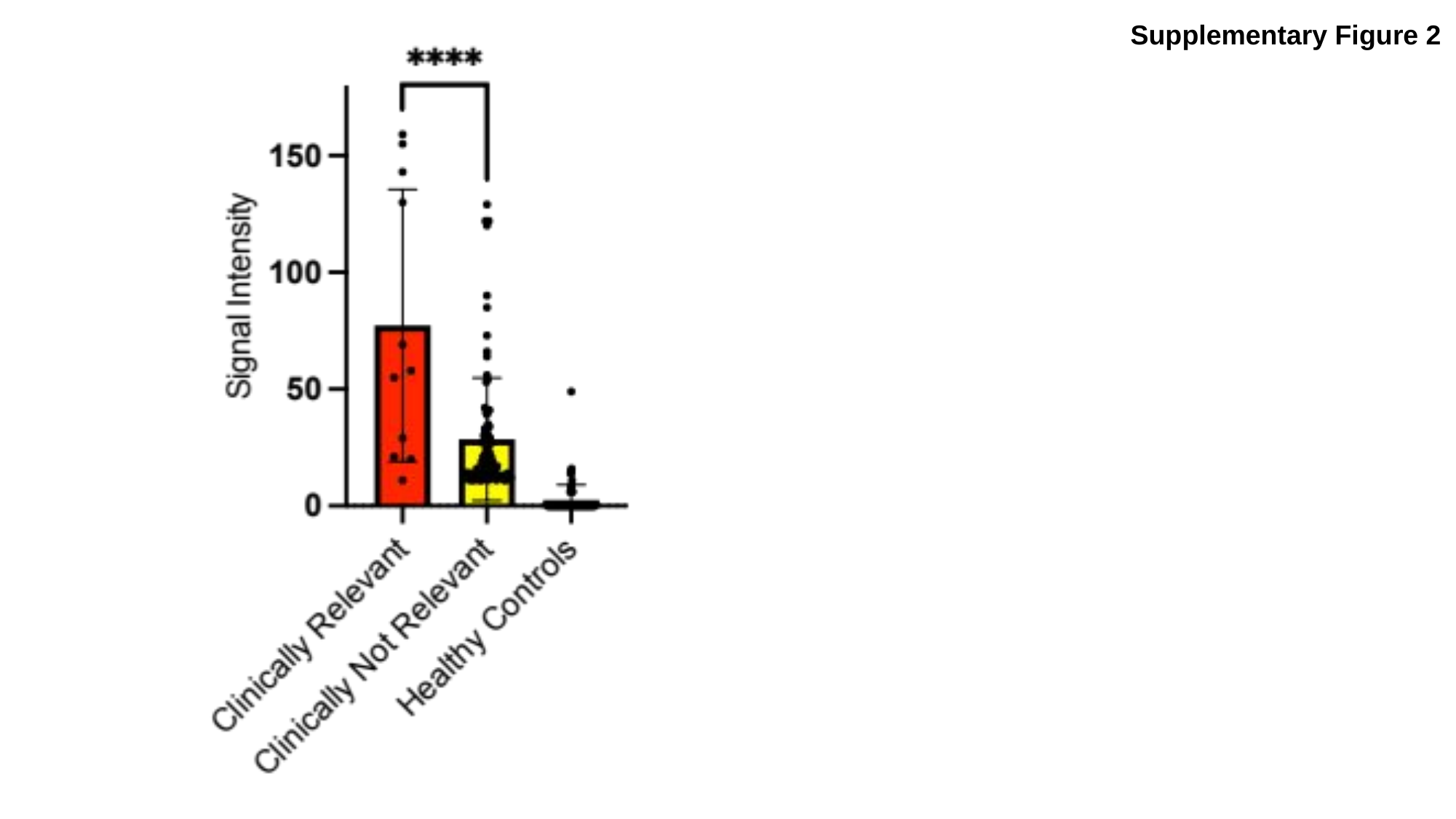

Supplementary Figure 2

Supplement: SUPPLEMENTARY FIGURE S1 — Prozone effect assessment example in a patient with positive Zic4 antibody on paraneoplastic neurological syndromes line immunoblot (Signal Intensity 32; ++ strong positive band) and CSF (Signal Intensity 90; +++ very strong positive band). CSF dilution (1:10) with indirect immunofluorescence findings for cerebellum (a), cerebrum (b) and stomach (c) tissue substrates. Serum dilution (1:100) with IIF findings for cerebellum (d), cerebrum (e) and stomach (f) tissue substrates. CSF - cerebrospinal fluid. [file Presentation_1.pptx]
